# In Silico, In Vitro, and In Vivo Evaluation of Caffeine-Coated Nanoparticles as a Promising Therapeutic Avenue for AML through NF-Kappa B and TRAIL Pathways Modulation

**DOI:** 10.3390/ph16121742

**Published:** 2023-12-18

**Authors:** Muhammad Hamid Siddique, Sidra Bukhari, Inam Ullah Khan, Asiya Essa, Zain Ali, Usama Sabir, Omiya Ayoub, Haleema Saadia, Muhammad Yaseen, Aneesa Sultan, Iram Murtaza, Philip G. Kerr, Mashooq Ahmad Bhat, Mariam Anees

**Affiliations:** 1 Department of Biochemistry, Quaid-i-Azam University, Islamabad 45320, Pakistan; hamidsiddique18@gmail.com (M.H.S.); sidrabukhari82@gmail.com (S.B.); iukhan@bs.qau.edu.pk (I.U.K.); asiyaessa78@gmail.com (A.E.); xainsolangee@gmail.com (Z.A.); usamasabir93@gmail.com (U.S.); omiyaayoub1812@gmail.com (O.A.); haleemababar700@gmail.com (H.S.); aneesa@qau.edu.pk (A.S.); irambch@qau.edu.pk (I.M.); 2 Institute of Chemical Sciences, University of Swat, Charbagh 19130, Pakistan; muhammadyaseen.my907@gmail.com; 3 School of Dentistry and Medical Sciences, Charles Sturt University, Sydney, NSW 2678, Australia; philip.kerr@gmail.com; 4 Department of Pharmaceutical Chemistry, College of Pharmacy, King Saud University, Riyadh 11451, Saudi Arabia; mabhat@ksu.edu.sa

**Keywords:** acute myeloid leukemia, docking, caffeine, MSNPs, nanomedicine, doxorubicin

## Abstract

Background: Advancements in nanoscience have led to a profound paradigm shift in the therapeutic applications of medicinally important natural drugs. The goal of this research is to develop a nano-natural product for efficient cancer treatment. Methods and Results: For this purpose, mesoporous silica nanoparticles (MSNPs) were formulated, characterized, and loaded with caffeine to develop a targeted drug delivery system, i.e., caffeine-coated nanoparticles (CcNPs). In silico docking studies were conducted to examine the binding efficiency of the CcNPs with different apoptotic targets followed by in vitro and in vivo bioassays in respective animal models. Caffeine, administered both as a free drug and in nanomedicine form, along with doxorubicin, was delivered intravenously to a benzene-induced AML model. The anti-leukemic potential was assessed through hematological profiling, enzymatic biomarker analysis, and RT-PCR examination of genetic alterations in leukemia markers. Docking studies show strong inter-molecular interactions between CcNPs and apoptotic markers. In vitro analysis exhibits statistically significant antioxidant activity, whereas in vivo analysis exhibits normalization of the genetic expression of leukemia biomarkers STMN1 and S1009A, accompanied by the restoration of the hematological and morphological traits of leukemic blood cells in nanomedicine-treated rats. Likewise, a substantial improvement in hepatic and renal biomarkers is also observed. In addition to these findings, the nanomedicine successfully normalizes the elevated expression of GAPDH and mTOR induced by exposure to benzene. Further, the nanomedicine downregulates pro-survival components of the NF-kappa B pathway and upregulated P53 expression. Additionally, in the TRAIL pathway, it enhances the expression of pro-apoptotic players TRAIL and DR5 and downregulates the anti-apoptotic protein cFLIP. Conclusions: Our data suggest that MSNPs loaded with caffeine, i.e., CcNP/nanomedicine, can potentially inhibit transformed cell proliferation and induce pro-apoptotic TRAIL machinery to counter benzene-induced leukemia. These results render our nanomedicine as a potentially excellent therapeutic agent against AML.

## 1. Introduction

The term “cancer” encompasses a range of conditions where cells multiply uncontrollably and invade surrounding tissues [1]. In the realm of cancer, leukemia represents a pathological condition whereby the hematopoietic cells in the bone marrow undergo malignant transformation, resulting in the excessive proliferation of leukocytes, or white blood cells [2]. This results in enhanced levels of immature white blood cells in the bloodstream, with decreased production of mature erythrocytes, lymphocytes, and platelets. Clinically, the presence of blast cells with a cell morphology of 30% or greater in the hematological analysis confirms the diagnosis of acute myeloid leukemia (AML) [3]. In the management of AML, conventional therapies for AML include chemotherapy, radiation, surgery, hormonal therapy, and immunotherapy.

Chemotherapy can induce immunogenic cell death in addition to the intended therapeutic effects. Many clinical trials have used natural products as key sources of anticancer medications, including paclitaxel and vincristine, amongst others. Despite the availability of these medications for the treatment of cancer, chemotherapy failure is a regular occurrence because of toxicities that are dose-restricting and the establishment of drug resistance. The utilization of nano-delivery systems in this regard enables the direct targeting and delivery of specific drugs to malignant regions, leading to an augmented permeability and intracellular accumulation of anticancer medications [4].

Doxorubicin is a known anti-leukemic drug that induces apoptosis by causing DNA damage in transformed cells by intercalating with DNA bases, causing the production of reactive oxygen species (ROS) [5]. The production of reactive oxygen species (ROS) by doxorubicin plays a key role in its cytotoxic effect, making it an effective treatment for cancer cells. However, its non-specific nature leads to various undesirable side effects, including hair loss, fatigue, vomiting, nausea, and diarrhea [6]. Hence, such drugs need to be replaced by natural products with potent anticancer efficacy.

Caffeine, a naturally occurring trimethylxanthine, exerts strong stimulating effects on the central nervous system of humans [7]. It promotes extrusion through efflux pumps, which are regulated by the transcription factor Pap1, ultimately facilitating detoxification processes [8] Caffeine is also known to have anticancer potential; however, its short half-life prevents it from being a drug of choice [9]. Caffeine is reported on for its ability to interfere with DNA repair mechanisms in cancer cells, making them more susceptible to cell death. It also interferes with the cell cycle progression of cancer cells, leading to cell cycle arrest, impeding the uncontrolled division of cancer cells [8].

Nanotechnology is gaining prominence as a promising field for therapeutic applications, and nanoparticles combined with caffeine show potential as an effective drug delivery system [10]. Nano-formulations in medicine are developing swiftly to increase drug uptake and delivery to specific sites, especially by the use of mesoporous silica nanoparticles (MSNPs) [11].

Due to the characteristics of caffeine and MSNPs, we developed caffeine-coated nanoparticles as a nanomedicine (CcNPs) and investigated its efficacy against benzene-induced AML in rat models through in silico, in vitro, and in vivo analysis against various anticancer markers. Hematologic profiling, renal and hepatic biomarker profiling, and underlying pathways analysis further demonstrate its potential as an effective therapeutic approach.

## 2. Results

### 2.1. Characterization of MSNPs and Drug Loading

Scanning electron microscope images reveal mono-distributed spheres of MSNPs with an average diameter of 120 nm (Supplementary Figure S1). The X-ray diffraction pattern confirms the formation of MSNPs, indicated by a significant diffraction peak at 2θ = 29.18 corresponding to MSNPs with a diameter of 118.18 nm. FTIR spectroscopy shows characteristic peaks of the nanomedicine like that of caffeine, indicating the coating of caffeine on the nanomedicine. The UV-Vis spectroscopy absorption patterns of caffeine, nanomedicine, and MSNPs confirm the loading of caffeine onto the nanoparticles, with a loading efficiency of 28% (Supplementary Figures S2–S4).

### 2.2. Nanomedicine Exhibiting Strong Interaction with Pro-Apoptotic TRAIL-DR5 Complex

**Figure 1:**
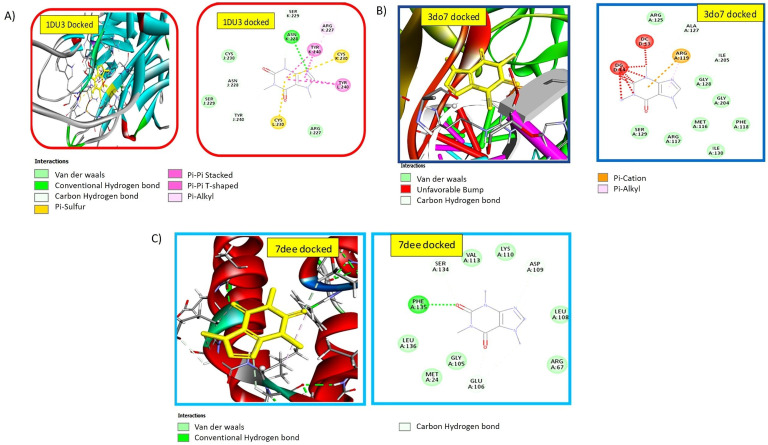
Molecular docking between ligand and (**A**) TRAIL-DR5 complex (PDB:1du3), (**B**) NFκB p52/RelB/DNA protein complex (pdb:3do7), (**C**) cFLIP-DISC complex (pdb:7dee) proposing strong binding affinities with anti-leukemic markers.

The TRAIL-DR5 protein’s binding site was docked with the caffeine in nanomedicine form (ligand) (pdb:1du3) as shown in Figure 1A. The binding affinity of the lowest value confirmation was found −6.8 and RMSD = 0.00 using PyRx. Further analysis using BIOVIA Discovery Studio showed the presence of hydrogen bonds, hydrophobic interactions, and Van der Waals interactions. Notably, conventional hydrogen bond interactions were observed between the ligand and the receptor amino acid ASN228. Additionally, four carbon–hydrogen bonds were detected between the ligand and the amino acids ASN228, SER229, and TYR240. Two pi–sulfur interactions with the amino acid CYS230 were also observed. Two pi–pi stacked interactions, two pi–pi T-shaped interactions with TYR240, and a pi–alkyl interaction with ARG227 were discovered among the five hydrophobic interactions. Various amino acid residues exhibited Van der Waals interactions as well.

### 2.3. Docking Studies Confirmed the Strong Binding Efficiency of the Ligand with Anti-Proliferative NF-kB p52/RelB/DNA Complex

The binding affinity between caffeine and the binding site of the NF-kB p52/RelB/DNA complex protein was determined to be −5.6 Kcal/mol, with an RMSD of 0.00, indicating excellent binding efficiency and interaction as in Figure 1B. Further analysis of this confirmation performed using BIOVIA Discovery Studio (version 2021) showed Van der Waals’ interactions, hydrophobic bonds, and hydrogen bonds. The interactions were also observed with DNA present in a protein complex. Notably, a significant interaction was observed with the amino acid ARG:119. These docking studies exhibited the binding of the nanomedicine with pro-apoptotic and anti-proliferative complexes, which was further confirmed via in vitro and in vivo studies.

### 2.4. Nanomedicine Exhibiting Strong Interaction with c-FLIP

The ligand was docked in the active site of c-FLIP (pdb:7dee) as shown in Figure 1C. There was a −5.3 binding affinity and an RMSD of 0.00. In addition to Van der Waals’ interactions, carbon–hydrogen bonds and conventional carbon–hydrogen bonds were also seen with amino acid residues.

### 2.5. Increased Antioxidant Potential of Caffeine in the Form of Nanomedicine

**Figure 2:**
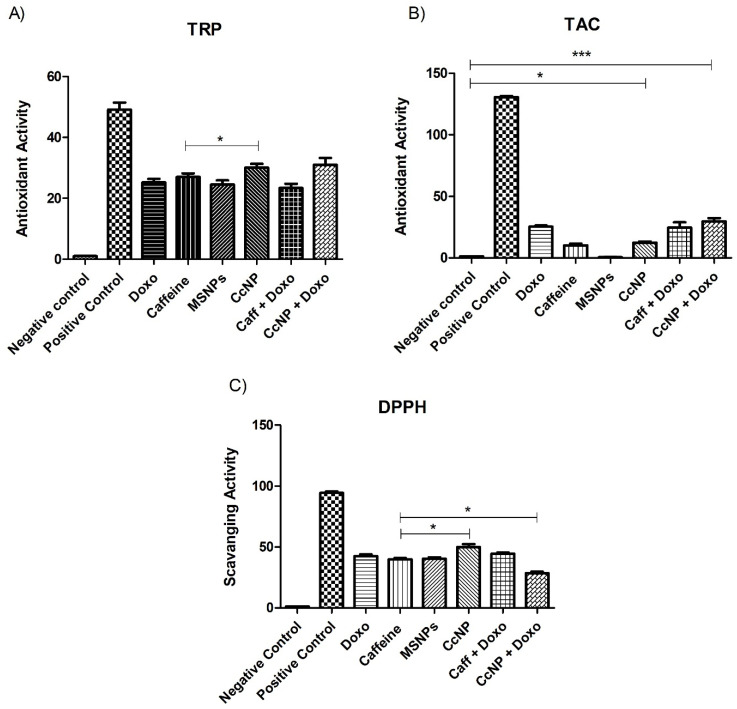
Antioxidant potential analysis of different treatment groups via (**A**) total reducing power assay, (**B**) total antioxidant capacity, and (**C**) 2,2-diphenyl-1-picrylhydrazyl (DPPH) assay. All treated groups exhibit significant antioxidant potential, especially the CcNP-treated group. Statistical significance levels are defined as * = *p* < 0.05; *** = *p* < 0.001.

The total reducing potential assay exhibits significant (*p* < 0.001) reducing activity in all treatment regimens. It is observed that the reducing potential of caffeine increases (*p* < 0.05) when administered in the form of nanomedicine as shown in Figure 2A. The total antioxidant capacity assay also exhibits a similar pattern. However, treatment with nanomedicine plus drug increased (*p* < 0.001) antioxidant activity by 16.5% compared to drug only (Figure 2B). Likewise, the DPPH assay confirmed a 24.8% increase (*p* < 0.05) in the antioxidant potential of caffeine as a nanomedicine (Figure 2C).

### 2.6. Enhanced Biological Potential of Nanomedicine as Anti-Depressant, Analgesic, and Anti-Coagulation Agent in Rat Models

**Figure 3:**
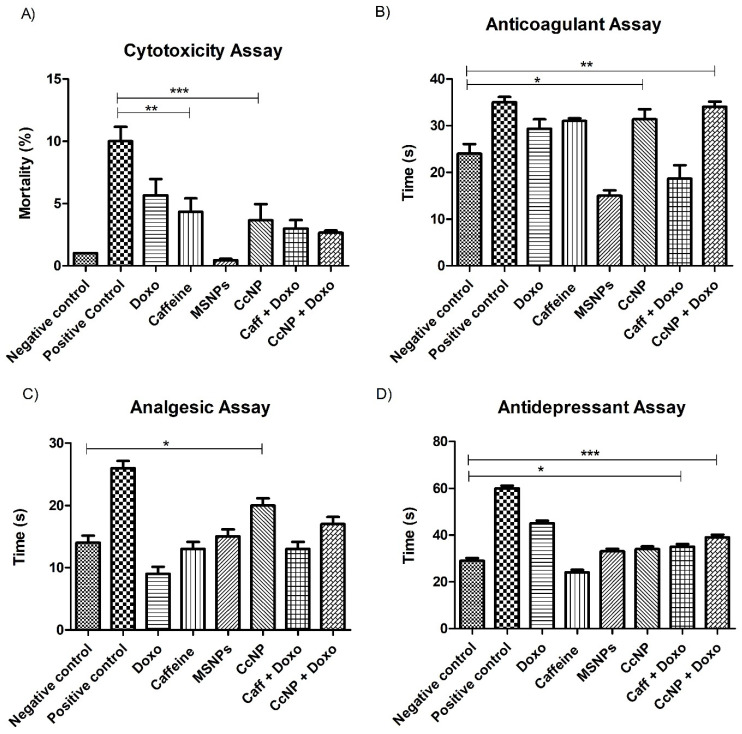
Analysis of the biological potential of different treatment regimes. (**A**) Brine shrimp assay exhibits reduced toxicity of nanomedicine (*p* < 0.05) as compared to chemotherapy. (**B**) The anti-coagulation potential of chemotherapy significantly increased (*p* < 0.01) when combined with nanomedicine. (**C**) The analgesic potential of caffeine significantly increased (*p* < 0.05) when used as nanomedicine (**D**) Nanomedicine exhibited significantly increased (*p* < 0.05) anti-depressant potential as compared to caffeine. Statistical significance levels are defined as * = *p* < 0.05; ** = *p* < 0.01; *** = *p* < 0.001.

The combination of nanomedicine with chemotherapy shows a decrease (*p* < 0.01) in cytotoxicity, as demonstrated by the brine shrimp assay (Figure 3A). Additionally, the combination therapy exhibits noteworthy improvements in the anti-depressant (15.3%), analgesic (88%), and anti-coagulation potential (16%) of nanomedicine (*p* < 0.001) compared to doxorubicin, confirming the bio-potency of nanomedicine in Sprague-Dawley rat models (Figure 3B–D).

### 2.7. Restoration of Liver, Heart, and Kidney Weights back to Normal after CcNP Treatment

Relative organ weight assessment showed an increase in weight of the liver (*p* < 0.05), heart (*p* < 0.001), and kidney (*p* < 0.05) in the benzene-treated groups (Supplementary Figure S5A–C). After treatment with nanomedicine (CcNPs), organ weights were reduced (*p* < 0.05) and were comparable to weights in the normal group. Similar results were obtained for treatment with MSNPs, doxorubicin alone, and the combination of CcNPs and doxorubicin.

### 2.8. Restoration of Blood Parameters upon Nanomedicine Administration

**Figure 4:**
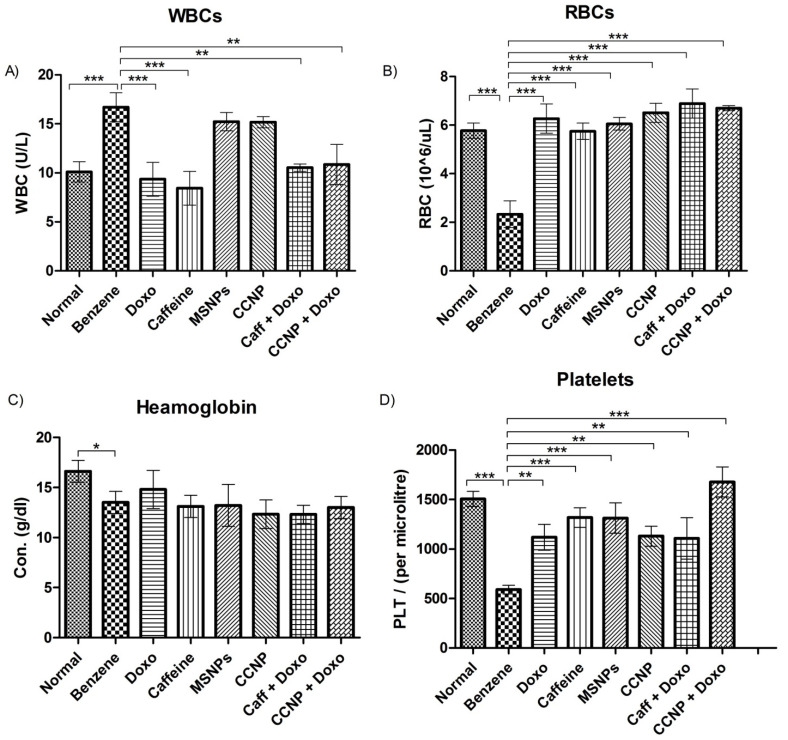
Blood parameters of different experimental groups. (**A**) Total white blood cells count, (**B**) red blood cells, (**C**) hemoglobin, (**D**) total platelets count. The values are shown as mean ± SEM. The intergroup comparisons were made using one-way ANOVA with Tukey’s post hoc test. Statistical significance levels are defined as * = *p* < 0.05; ** = *p* < 0.01; *** = *p* < 0.001.

Morphological studies exhibited erythrocytosis and the presence of leukoblasts in the benzene-administered leukemic rats. The nanomedicine treatment restored the normal morphology of cells, exhibiting intact red blood cell morphology and a normal nucleus-to-cytoplasm ratio in the treated groups (Supplementary Figure S6). Whole blood count analysis exhibited an abnormal increase in total WBCs and a significant decrease in RBCs in the benzene-treated rats (*p* < 0.05). In the case of WBCs, the combinational therapy gives more significant results than the nanomedicine alone, with the combination of nanomedicine and doxorubicin proving beneficial. (Figure 4A).

Levels of monocytes, eosinophils, neutrophils, and lymphocytes also increased to abnormal levels in the leukemic rats and were positively impacted by nanomedicine. The observed effect is not statistically significant. On the other hand, RBC counts were restored to normal upon nanomedicine treatment (Figure 3B). Hemoglobin levels and platelet counts were also reduced in the leukemic group (*p* < 0.05) but the treatment did not show a significant impact on hemoglobin levels; however, the platelets improved significantly (*p* < 0.05) among the treated rats with varying degrees of efficacy (Figure 4C,D).

### 2.9. Enzymatic Activities Normalized after Drug Treatment

**Figure 5:**
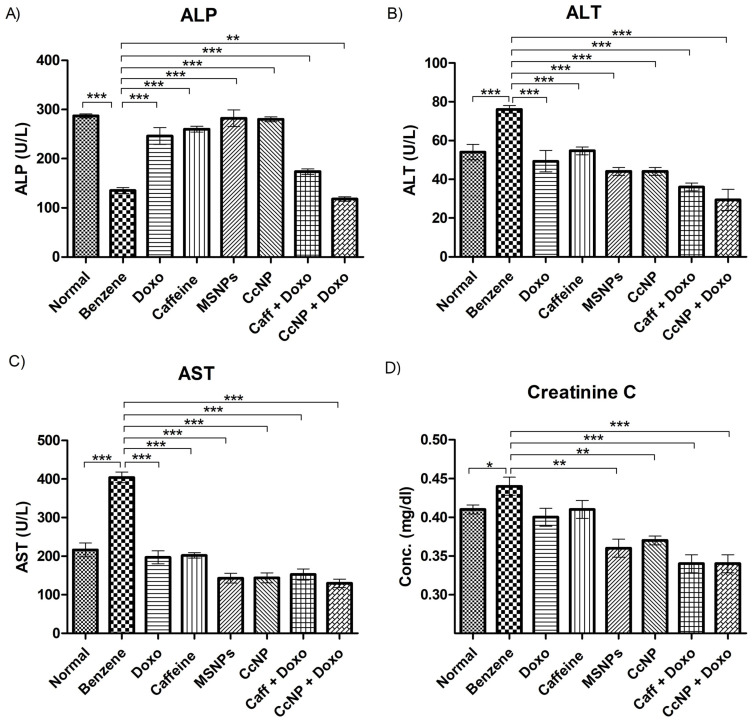
Levels of hepatic and renal biomarkers among various experimental groups. (**A**–**D**) exhibit the mean + SEM values of ALP, ALT, AST, and creatinine, respectively. The intergroup comparisons were made using one-way ANOVA with Tukey’s post hoc test. Statistical significance levels are defined as * = *p* < 0.05; ** = *p* < 0.01; *** = *p* < 0.001.

Hepatic biomarkers showed decreased (*p* < 0.001) ALP levels and increased (*p* < 0.001) levels of ALT and AST in benzene-treated rats, which were restored to normal upon administration of nanomedicine. ALP levels showed a marked increase (*p* < 0.001), while ALT and AST exhibited a significant decrease (*p* < 0.001) upon nanomedicine treatment. Other therapeutic regimes also showed promising results with variable efficacy (Figure 5A–C). Administration with nanomedicine effectively recovered (*p* < 0.01) the normal creatinine levels that were increased significantly (*p* < 0.05) in benzene-treated rats showing renal malfunction. The combination treatment regimens showed even better results in improving renal function (Figure 5D).

### 2.10. Suppression of STMN1 Expression by Nanomedicine

**Figure 6:**
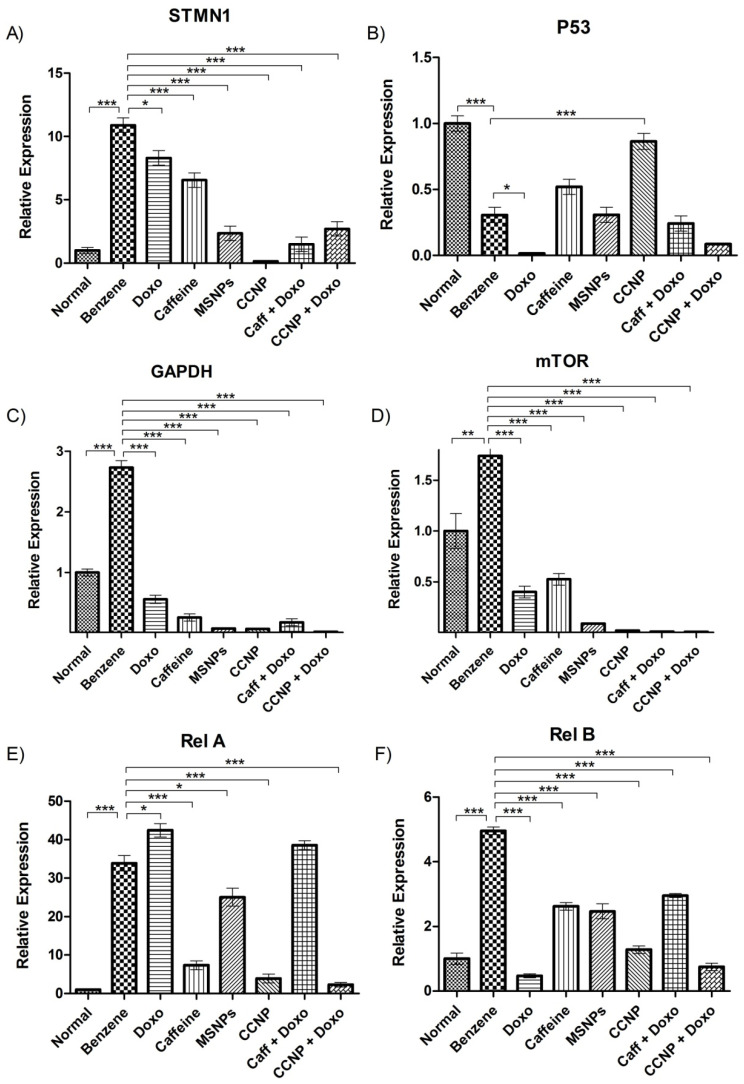
Gene expression analysis of molecular markers among various experimental groups. (**A**) depicts AML induction via assessment of STMN1. (**B**) exhibits tumor suppressor gene P53. (**C**,**D**) reveal GAPDH and mTOR expression. (**E**,**F**) represent NF-kappaB pathway markers Rel A and Rel B. The values are shown as mean ± SEM. The intergroup comparisons were made using one-way ANOVA with Tukey’s post hoc test. Statistical significance levels are defined as * = *p* < 0.05; ** = *p* < 0.01; *** = *p* < 0.001.

Acute myeloid leukemia (AML) is characterized by the overexpression of STMN1, which serves as a genetic marker for the disease. The benzene-treated group exhibited a significant increase in STMN1 expression (*p* < 0.001), confirming AML induction. However, treatment with the nanomedicine led to a significant reduction in STMN1 expression (*p* < 0.001) (Figure 6A).

### 2.11. Upregulation of Tumor Suppressor Gene p53 by Nanomedicine

The guardian of the genome, p53, mainly works as a tumor suppressor gene and its expression is often altered during the malignancy onset. The normal genetic expression of p53 was restored upon treatment with nanomedicine (*p* < 0.001), subsequent to decreasing upon AML induction in our leukemic rat model (Figure 6B).

### 2.12. Regulation of Glycolysis by Nanomedicine in Leukemic Rats

GAPDH (Glyceraldehyde 3-phosphate dehydrogenase), being a critical enzyme regulating the sixth step of glycolysis, is a molecular indicator of energy production in the cell. There was a significant increase in the genetic expression of GAPDH after benzene injections, indicating excessive energy production, ensuring proliferation. However, the nanomedicine significantly restored the expression of GAPDH to normal (*p* < 0.001). Caffeine, doxorubicin, and other drug combinations also showed positive results (Figure 6C).

### 2.13. Regulation of the mTOR Pathway by Nanomedicine

mTOR is a serine/threonine protein kinase that is involved in the regulation of transcription, cell growth, proliferation, and survival. The expression of mTOR was highly increased because of benzene induction, but significantly reduced (*p* < 0.001) upon nanomedicine administration. The drug combinations with chemotherapy also proved very beneficial (*p* < 0.001), as shown in Figure 6D.

### 2.14. Nanomedicine Induces Anti-Proliferative Effects through NF-Kappa B Pathway Inhibition

One of the major pathways regulating the proliferation of somatic cells in acute myeloid leukemia is the NF-kappa B pathway. The expression of NF Kappa B pathway genetic markers Rel-A and Rel-B increased significantly in leukemic rats, indicating over-proliferation. Treatment with the nanomedicine alone and in combination with doxorubicin significantly reduced the expression of Rel-A (*p* < 0.001). Rel-B expression was significantly reduced by doxorubicin, nanomedicine, and a combination (*p* < 0.001), thus pointing towards an inhibition of the non-canonical pathway (Figure 6E,F).

### 2.15. Regulation of TRAIL Pathway by CcNP

**Figure 7:**
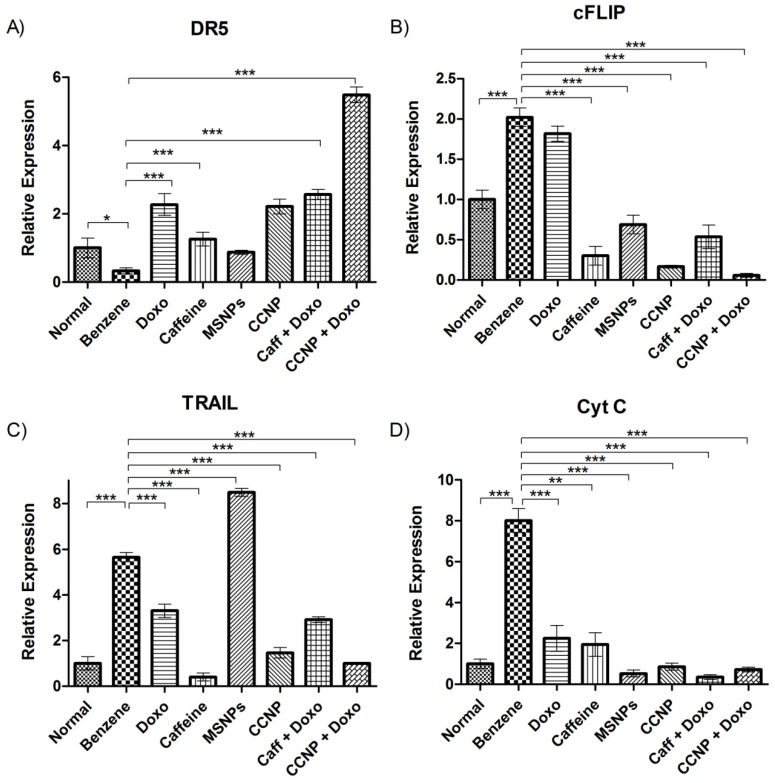
Relative gene expression of TRAIL pathway components including death receptor 5 (**A**), FLICE-like inhibitory protein (**B**), TRAIL ligand (**C**), and cytochrome-c (**D**). The values are shown as mean ± SEM. The intergroup comparisons were made using one-way ANOVA with Tukey’s post hoc test. Statistical significance levels are defined as * = *p* < 0.05; ** = *p* < 0.01; *** = *p* < 0.001.

Benzene downregulates the expression of death receptor 5 (DR5) and upregulates the inhibitory protein FLIP, both of which play a crucial role in apoptosis regulation via the extrinsic TNF-related apoptosis-inducing ligand (TRAIL) pathway. It is observed that CcNP not only restored the DR5 expression (*p* < 0.001), but also conjugated with the classical chemotherapy to give a synergistic effect in upregulating its expression (*p* < 0.001). Additionally, CcNP downregulated the anti-apoptotic protein cFLIP (*p* < 0.001), thereby lifting the inhibition of apoptosis (Figure 7A,B). The expression of the TRAIL ligand was increased by benzene (*p* < 0.001) but the nanomedicine reduced the levels back to normal (Figure 7C). Cytochrome c levels increased upon benzene induction, probably due to mitochondrial membrane damage in leukemic cells (Figure 7D). The levels were significantly restored by various drug regimens including nanomedicine (*p* < 0.001). The nanomedicine, however, did not exhibit a significant effect on the expression profiles of caspases 3, 7, 8, and 9.

## 3. Discussion

Nanoparticles are being extensively employed as drug delivery vehicles because of their ability to minimize off-target side effects by crossing blood–brain and cell membrane barriers, thereby overcoming the major obstacle of site-specific delivery [12]. In this study, scanning electron microscopy confirmed the hollow spherical morphology of MSNPs along with particle size. XRD data further verified the particle size and shape, which corresponds to the data already published [13]. The MSNP peaks confirmed the presence of SiO_2_ and reconfirm the previously published studies [14]. Both FTIR and UV analyses yielded similar outcomes, confirming the successful coating of caffeine onto the MSNPs [15].

In silico simulations were performed to analyze the interaction of nanomedicine with the active site of target proteins/complexes. As FTIR results confirmed the availability of caffeine on the outer surface of the nanomedicine particles, its interactions with active site residues were assessed using caffeine as substrate. Earlier investigations have reportedly revealed that quinacrine (QC) i.e., a known substrate, induces cellular apoptosis by modulating both the TRAIL-DR5 complex and the mitochondrial intrinsic pathway [16]. The binding affinity and interactions of QC and caffeine were compared. The binding affinity values were found to be −6.5 for QC and −6.8 for caffeine. The interactions of caffeine with the active site of the target complex included multiple hydrogen bonds, which were more than those of QC. Moreover, QC attaches itself to the edge of the protein complex and forms a QC-TRAIL-DR5 complex. Caffeine also enters the protein complex and forms a caffeine-TRAIL-DR5 complex. Likewise, caffeine exhibited strong hydrogen bond interaction with the TRAIL-DR5 complex due to its exposed hydrophilic ends. The Van der Waals’ interactions observed were due to the exposed non-polar ends (alkyl groups) of the caffeine, whereas pi–pi stacked and pi–pi T-shaped interactions were due to the aromatic rings of the ligand. The multiple hydrogen bonding interactions suggests that caffeine acts as a promotor of the TRAIL-DR5 complex. These multiple interactions of caffeine with proteins are consistent with the CcNP-TRAIL-DR5 complex being much more stable than the QC-TRAIL-DR5 complex.

Another target complex, i.e., the p52:RelB:kB DNA, identifies the DNA backbone becoming asymmetrical after making direct contact with the protein through ARG 125. Earlier in vitro binding studies and cell-based reporter assays have indicated that the presence of Arg 125 in the p52:RelB complex is indispensable for the binding and subsequent activation of transcription at selective kB sites, but not all [17]. Our docking studies reveal the interaction of ARG 119 and ARG 125 with caffeine, which results in steric incompatibility with the DNA at the ligand’s binding site, and thus the caffeine in the form of nanomedicine acts as competitive inhibitor which, in turn, confirms the inhibitory action of the ligand (caffeine) and thus prevents complex formation.

The cellular FLICE-like inhibitory protein (c-FLIP), a procaspase-8-like molecule, has been identified as a key regulator of apoptosis triggered by death ligands. It achieves this by binding to FADD and/or caspase-8 or -10 in a ligand-dependent manner, effectively inhibiting apoptosis induced by tumor necrosis factor-alpha (TNF-alpha), Fas-L, and TRAIL. This inhibitory action prevents the formation of the death-inducing signaling complex (DISC) and subsequently hinders the activation of the apoptotic cascade [18]. Dapagliflozin, according to recent studies, causes apoptosis by downregulating cFLIP-L and increasing cFLIP-S instability and may be a viable therapeutic candidate for the treatment of human kidney cancer [19].

Based on this finding, we docked caffeine in c-FLIP. Although dapagliflozin’s binding affinity was found to be lower (−6.5) than that of caffeine, the interactions made by dapagliflozin were Van der Waals’ and pi–alkyl interactions, whereas those made by caffeine were hydrogen bonds in addition to Van der Waals’ interactions and showed more resemblance to interactions made by Procaspase-8, so caffeine is anticipated to be a good downregulator of cFLIP, which was confirmed by relative expression analysis.

The increase in organ weights observed during in vivo experimentation was potentially due to the effect of benzene metabolites, which target these organs directly and result in free radical formation associated with hypertrophy [20]. However, the swift recovery in organ weights by all the treatment regimens provides significant evidence of the protective effect of nanomedicine against benzene-induced hypertrophy.

Benzene induces acute myeloid leukemia as a result of its metabolites, which include phenol, catechol, and hydroquinone. Morphological analysis reveals that benzene, via these cytotoxic metabolites, exhibits erythrocytosis, leading to a reduced total RBC count along with other abnormalities like eosinophilia and leukocytosis [21]. Likewise, due to multiple mutations caused by benzene in bone marrow cells affecting the differentiation of leukocytes, the total WBC count gets elevated, resulting in increased levels of immature leukoblasts in leukemic blood [22]. Similar observations were made in our study as well. Administering different drug regimens restored a normal blood profile in leukemic rats significantly by downregulating erythrocytosis, eosinophilia, and leukocytosis [21]. Caffeine is known to induce G_2_/M arrest and apoptosis in leukemic cells [23]. The observed reduction in platelet levels in our study is likely attributed to the detrimental effects on bone marrow cells caused by benzene inoculation. Notably, treatment with nanomedicine significantly increased platelet counts by downregulating thrombocytopenia [24].

Benzene also dysregulates normal levels of ALP, ALT, and AST due to ROS production by its secondary metabolites, as the liver is one of the target organs of benzene toxicity [23] ALP levels in benzene-induced leukemic rats decreased due to the lack of detectable leukocyte alkaline phosphatase (LAP) mRNA in AML. Whereas, the elevation in ALT levels was potentially due to the abnormal proliferation of liver cells leading to increased alanine transaminases in leukemic rats [25]. Likewise, AST concentrations in blood serum also increased as an indicative of hepatocytotoxicity and benzene-induced liver cell damage. Benzene administration also elevated serum creatinine levels, predicting acute kidney injury in leukemic rats [26]. However, such abnormal levels of these hepatic enzymes and creatinine were significantly restored to normal by our nanomedicine, probably due to decreased cytotoxicity and increased antioxidant potential.

Genetic expression analysis was performed by homogenizing liver cells, since the liver is the metabolizing hub for almost all xenobiotic chemicals like benzene. STMN1, being one of the most over-expressed genes in AML, showed elevated expression levels in benzene-administered leukemic rats, confirming leukemia induction at the genetic level [27]. Treatment with CcNP minimized the benzene effect by decreasing STMN1 expression, thereby potentially altering the actin function and microtubule dynamics of cells [28].

Loss of P53, a common tumor suppressor, is a prevalent genetic alteration observed in cancers. In AML, P53 expression becomes deregulated due to mutations and ROS-induced DNA damage. However, our nanomedicine restored altered P53 levels in leukemic rats, presumably through its pro-apoptotic and antioxidant properties. Additionally, caffeine’s inhibitory potential against lipid peroxidation induced by reactive oxygen species may have played a role in this restoration, as reported by [29].

Overexpression of GAPDH in the benzene-treated group was potentially a result of enhanced glycolysis, which is also a known cancer hallmark [1,30]. However, treatment with our nanomedicine and other regimes restored normal GAPDH expression levels by recovering cellular energetics and inhibiting over-proliferation in transformed cells [31].

mTOR, which is a central intracellular and extracellular signal processor for many key cellular mechanisms, such as growth, proliferation, and survival, was also observed to be increased in the benzene-treated groups. It also regulates leukemic cell growth, tumor-associated angiogenesis, and the expression of vascular endothelial growth factor [32]. This is due to the known ability of the mTOR protein to recruit micro RNAs for silencing apoptotic genes, leading to leukemogenesis [33]. Our nanomedicine significantly decreased mTOR levels, probably due to its pro-apoptotic potential.

Rel A and Rel B from the NF-kappa B pathway sustain cell survival in AML as depicted by their increased mRNA transcripts in leukemogenesis [34]. Doxorubicin, being an apoptosis inducer via ROS production, decreased Rel B levels [35]. However, in the case of Rel A, DNA damage induced by doxorubicin further activated the canonical NF-κB pathway, leading to doxorubicin resistance in leukemic cells, which might be the reason behind high levels of Rel A expression (unlike Rel B) in the doxorubicin-treated rats. However, the downregulation of the NF-kappa B pathway markers, both Rel A and Rel-B, by our nanomedicine individually and in combination suggests its anti-proliferative and anti-tumor potential in leukemic rats. This is probably because of the known anti-survival and inhibitory potential of caffeine against Rel A and Rel B, which might be enhanced in the form of nanomedicine as MSNPs increased the bioavailability, biostability, and half-life of coated caffeine as previously reported [36].

To assess the role of apoptosis, the TRAIL pathway has been one of the most explored mechanisms. Increased expression of the TRAIL ligand in benzene-treated rats is not surprising, as many benzene derivatives are known to be involved in TRAIL-mediated cell death [37]. Over-expression of cFLIP inhibits the TRAIL activity in such leukemic rats [38]. The significantly enhanced expression of the TRAIL ligand and decreased cFLIP suggest our nanomedicine as a potent TRAIL pathway regulator and apoptosis inducer. This is further evident from the increased expression of DR5 in the nanomedicine-treated group. The combination of nanomedicine with chemotherapy further increased the DR5 expression levels, pointing towards some cross-linking mechanism [39]. An increase in DR5 in response to therapy is an effective step towards TRAIL-induced apoptosis through extrinsic pathways. But merely the upregulation of death receptor(s) is not enough to induce apoptosis; their functional trimerization is also necessary for the induction of cell death. Since we did not see any upregulation of initiator or effector caspases, we suggest the use of some agonistic antibody to increase the functional efficacy of death receptors to induce apoptosis in future studies [40]. Elevated levels of cytochrome-c in leukemic rats indicate its rapid production and release from mitochondria owing to mitochondrial membrane damage and cell death. However, treatment with various drug regimens restored the normal levels, probably due to antioxidant and other protective effects.

## 4. Materials and Methods

### 4.1. Synthesis of Mesoporous Silica Nanoparticles (MCM-41 Generation)

Mesoporous silica nanoparticles were synthesized using a chemical method [41]. The pH of deionized water (442 mL) was kept at 11 by adding 29% ammonium hydroxide (Sigma-Aldrich Cat. No. 1336-21-6; EMSURE®, Darmstadt, Germany) dropwise. The solution was heated to 50 °C, and 0.279 g of cetyltrimethylammonium bromide (CTAB) (Sigma-Aldrich Cat No. 57-09-0; Calbiochem®, Darmstadt, Germany) was added while stirring continuously. The pH was maintained at 10.3, and the solution was allowed to cool to room temperature. Next, tetramethyl orthosilicate (TMOS, 1.394 mL) (Sigma-Aldrich Cat. No. 78-10-4; Darmstadt, Germany) was added with constant stirring, resulting in turbidity after 2 min. The solution was left to stir overnight, followed by filtration and washing with deionized water. The obtained particles were dried at 90 °C and calcined at 500 °C for 5 h, yielding a white product. The product was finely ground into powder form. The dried powder was refluxed in methanol (100 mL) (Sigma-Aldrich Cat No. 67-56-1; Darmstadt, Germany) with concentrated HCl (1 mL) (Sigma-Aldrich Cat No. 7647-01-0; Darmstadt, Germany) for 24 h at 50 °C to remove the CTAB template. The resulting mesoporous silica nanoparticles (MSN) were isolated by filtration, extensively washed with water and methanol, and then dried at 100 °C for 24 h.

### 4.2. Characterization of Nanoparticles

The morphology and size of the mesoporous silica nanoparticles (MSNPs) were assessed using a field-emission scanning electron microscope (SEM, LEO SUPRA 55; Carl Zeiss AG, Oberkochen, Germany). For SEM analysis, a small portion of the powder was affixed to the SEM stage with carbon tape, and images were captured at an operating voltage of 12 keV. MSNPs were then further characterized through X-ray diffraction (XRD) analysis, UV/Vis spectroscopy and FTIR. XRD analysis was conducted on an X-ray spectrophotometer (Bucker D8 Advance, Karlsruhe, Germany), using Cu-Kά radiation (λ = 1.54 °A), operating at 40 KV and 30 mA. The size of nanoparticles was determined from full width at half maximum (FWHM) using the Debye-Scherrer equation (D = 0.9B/βCos) [42]. FTIR analysis was performed by loading liquified sample (400 μL) in an FTIR spectrometer (Bruker, Tensor 27; Ettlingen, Germany) with a scan range of 400 to 4000 cm^−1^, resolution of 4 cm^−1^. UV-Vis spectrophotometry (Perkin Elmer UV/Vis–Lambda 25; Shelton, CT, USA), was employed to obtain the optical absorption spectra of uncoated mesoporous silica nanoparticles (MSNPs), the drug alone, and the drug-loaded nanoparticles. The absorption spectra were measured at room temperature within the range of 250–800 nm.

### 4.3. Drug Loading

To prepare the drug-loaded nanomedicine, caffeine (0.583 g) (Sigma Aldrich Cat. No. C0750-5G; ReagentPlus®, Darmstadt, Germany) was mixed with normal saline (35 mL). The solution was subjected to sonication for 3 h, followed by the addition of MSNPs (145.75 mg) for overnight stirring. The percentage of loaded drug was calculated using a modified version of the cited method [43]. After preparing the nanomedicine, a 1 mL aliquot was centrifuged at 10,000 rpm for 10 min. Subsequently, the UV-Vis spectrum was recorded at a wavelength of 209 nm.

Drug Loading = W1/W2 × 100; Encapsulation Efficiency = W2 − W3/W2 × 100.

(W1 is the weight of caffeine in nanomedicine, W2 is nanomedicine, and W3 is the weight of the extract that remained in the supernatant). In order to enhance the bioavailability of the nanomedicine, polyethylene glycol (PEG, 0.006 g) (Sigma-Adrich Cat No. 25322-68-3; BioUltra, Darmstadt, Germany) was mixed in DMSO (4 mL) (Sigma-Adrich Cat No. 67-68-5; BioReagent, Darmstadt, Germany) to make a polymer solution [44]. The mixture was sonicated until the PEG was fully dissolved. Subsequently, the polymer solution was combined with the caffeine-loaded silica nanoparticles, up to a volume of 35 mL, and subjected to continuous stirring for two hours [45].

### 4.4. Molecular Docking Studies

The docking of the synthesized nanomedicine (ligand) to the active site of pro-apoptotic (cFLIP-DISC complex, TRAIL-DR5 complex) and anti-proliferative markers (NFkB-p52/Rel B/DNA complex) was achieved with the help of PyRx (v.0.8) (accessed on 18 February 2020) [46] and Autodock Vina (v.1.2.5) [47]. The ligand’s energy was minimized using Autodock, and the resulting protein–ligand interactions corresponded to the values predicted by quantum chemistry simulations [48]. The binding affinity values were examined, and these reflected the strength of the interactions between ligand and the targeted proteins. Additionally, BIOVIA Discovery Studio (v.4.5) [49] was used to display the 2D and 3D interactions of amino acid residues.

### 4.5. Cytotoxicity Assay

To determine the optimal dosage of the drugs, a brine shrimp assay was conducted. For this purpose, brine shrimp eggs were hatched in sea salt solution. Fifteen brine shrimp were counted under a microscope and placed in serial dilutions for 24 h as shown in Supplementary Table S1. Each concentration was replicated three times, and a blank control using distilled water was included. The lethal concentration for 50% mortality (LC_50_) after 24 h of exposure was determined using the probit method [50].

### 4.6. In Vitro Bioassays

The antioxidant potential was assessed by performing multiple assays. Total antioxidant capacity was calculated via the phosphomolybdenum method [51] with slight modifications. Likewise, total reducing power and DPPH assays were performed following the methods reported by Moein and coworkers [52].

### 4.7. In Vivo Bioassays

The pain-relieving potential of different treatment regimens was assessed using the hot plate assay [53]. The anti-coagulant assay, which determines the time required for blood to clot in the absence of any exotic substance, was performed [54]. Intraperitoneal dosing was administered to each group of mice, and the initial reading was taken immediately after dosing. For this, the tails of the mice were cleaned using ethanol and then 1–2 mm of the tip of the tail was cut using a sterilized pair of scissors. By squeezing the tail, a big drop of blood was placed onto a clean, microscope slide. The blood was observed using a toothpick drawn through it in circular motions until a fibrin thread was observed. The time of fibrin thread formation, i.e., coagulation, was measured using a stopwatch. Another reading was taken 3 h after dosing and the results were then analyzed. The negative control utilized DMSO (0.1%), while aspirin (1 mg/kg) served as the positive control. Furthermore, the anti-depressant activity was evaluated using the conventional tail suspension method described by Zhou and coworkers [34].

### 4.8. Experiment Design and Sprague-Dawley Model

The study was approved by the Institutional Review Board of Quaid-i-Azam University through Approval Letter No. #BEC-FBS-QAU2020-221. Forty female Sprague-Dawley rats were bought from the National Institute of Health (NIH), Islamabad, and divided into eight different groups, which were given different treatments as follows: Group (1) normal (saline); Group (2) benzene only; Group (3) doxorubicin + benzene; Group (4) caffeine + benzene; Group (5) MSNPs + benzene; Group (6) CcNPs + benzene; Group (7) caffeine + doxorubicin + benzene; Group (8) CcNPs + doxorubicin + benzene. These rats were given a seven-day acclimatization period and were housed at the Primate Facility of Quaid-i-Azam University, with a 12 h light/dark cycle and unrestricted access to water and rat feed.

Benzene injections were then prepared by mixing benzene (Sigma Aldrich Cat. No. 71-43-20; Darmstadt, Germany) and normal saline, keeping the aspect ratio 1:3. Benzene solution (200 μL) was injected into the tail vein of rats in groups 2–8 for 14 alternate days for leukemia induction followed by leukemia confirmation.

Doxorubicin (10 mg with 5 mL of normal saline), a chemotherapeutic drug, was purchased from Actavis, Italy (Cat. No. 25316-40-9), and a stock solution of 3.75 mg/1.8 mL was prepared. After 14 intravenous doses of benzene, a standard dose of doxorubicin (0.625 mg/0.3 mL) was given to rats in groups 3, 7, and 8 on alternate days for 3 weeks.

A stock solution of caffeine (Cat. No. C0750-5G) was prepared by mixing caffeine (0.583 g) in normal saline (35 mL). After confirmation of leukemia induction, the stock solution was administered (0.3 mL/rat) intravenously to rats in groups 4 and 7 for three weeks consecutively (8 mg/kg).

Meanwhile, caffeine-coated nanoparticles (CcNPs) were administered (0.3 mL/rat) to rats in groups 6 and 8 intraperitoneally for 3 weeks on alternate days. Except MSNPs, i.e., group 5, and caffeine-coated nanoparticles (CcNPs), i.e., groups 6 and 8, all chemicals administered including caffeine and doxorubicin were in form of free drugs.

After completing respective doses, all rats were dissected according to the guidelines given in the Guide for the Care and Use of Laboratory Animals [55].

### 4.9. Morphological Analysis

Blood slides were prepared after dissections and stained with Giemsa dye for morphological studies. The slides with blood smears were air-dried, and the dye was added dropwise onto them, allowing it to stain for 10 min. The stained slides were washed with tap water and air-dried again before microscopy examination [56].

### 4.10. Blood Profiling

To determine total blood cell count, the blood complete picture (CP) was calculated using an automated Z3 hematology analyzer (Zybio Inc. Shenzhen, China) at Islamabad Diagnostic Centre (IDC), Pakistan. This provided information on HGA, PLT, RBC, WBC, and other blood parameters. For the evaluation of hepatic and renal enzyme activity, a Micro Lab 300 auto-analyzer (Merk, Darmstadt, Germany) was used to perform biochemical assays. The parameters analyzed were alkaline phosphatase (ALP), alanine aminotransferase (ALP), aspartate aminotransferase (AST), and creatinine using biochemical analysis kits (AMP diagnostic kits).

### 4.11. mRNA Extraction

For relative expression analysis via real-time PCR, mRNA was extracted from the liver tissue. For this purpose, frozen tissue samples weighing 10 mg were taken and homogenized in 1 mL of TRIZOL (Sigma-Aldrich Cat No. 136426-54-5; Darmstadt, Germany). This sample was processed further using the protocol described by Chomczynski and co-workers [57]. The RNA extracted was then quantified by NanoDrop™ 2000/2000c Spectrophotometer (Cat. No: ND-2000; Waltham, MA, USA), and run on 1% agarose gel electrophoresis for quality assurance.

### 4.12. cDNA Synthesis

The mRNAs obtained were then projected to cDNA synthesis by using a VIVANTIS kit (cDSK01-050, Shah Alam, Malaysia). To verify the cDNA synthesis, conventional PCR was performed according to the manufacturer’s instructions to evaluate the annealing temperatures of designed primers along with cDNA confirmation. The amplification of the desired transcript by PCR was achieved by adding the reagents of the reaction mixture in 200 µL PCR tubes (Axygen, Union City, CA, USA). After PCR, the amplified product was visualized on 2% agarose gel along with ethidium bromide [58]. The size-specific amplicons were then confirmed.

### 4.13. RT PCR Expression Analysis

For relative expression analysis and quantification of amplified cDNA, MIC qPCR by BioMolecular Sciences (Sydney, Australia) was utilized. A non-specific binding dye, EvaGreen^®^ Master mix (Biotium, Inc., Fremont, CA, USA), was used during this procedure. Several genes were screened for relative gene expression analysis, including:

STMN1 (F-TTGCCAGTGGATTGTGTAGAG, R-TTCTTTTGATCGAGGGCTGAG),
P53 (F-TCCGACTATACCACTATCCACTAC, R-GCACAAACACGAACCTCAAAG),
GAPDH (F-TCCAGTATGACTCTACCCACG, R-CACGACATACTCAGCACCAG),
mTOR (F-AGTGAAAGTGAAGCCGAGAG), (R-CGACAAGGAGATAGAACGGAAG),
Rel A (F-CTACGAGACCTTCAAGAGCATC, R-GATGTTGAAAAGGCATAGGGC),
Rel B (F-CTTTTCTCAAGCTGACGTGC, R-AGATCTCCAGGTCCTCGTATG),
DR5 (F-TCAACCCTGTGCCAATCC, R-ATGAACTCCTTCCAGCGTG),
cFLIP (F-AGAAGCCCTCACCTTGTTTC, R-CTCTTGTCCTTGGCTACCTTG),
TRAIL ligand (F-CACATTACCGGGATCACTCG, R-AGCTCTCCGTTTCTCAAGTG)
and Cyt-c (F-CCCTAAGAGTCTGATCCTTTGTG, R-TCCAGTCTTATGCTTGCCTC).

The Pfaffl method was used to calculate the fold change in the gene expression of samples. Relative Expression Software Tool (REST-384, version 2 beta v.2, 2006) was used, calculating the relative expression in real-time PCR using pair wise fixed reallocation randomization test. Melt curve analysis was performed at the end to detect any non-specific products. q-PCR products under the melt curve graph showing a single peak denote the absence of primer dimers or non-specific products.

### 4.14. Statistical Analysis

Descriptive statistics were computed using GraphPad Prism software (version 5.01, 2007; Boston, MA, USA), and results were reported as mean ± standard error mean (SEM). Statistical significance was determined, and a *p*-value < 0.05 was considered to be statistically significant (<0.05 = *, <0.01 = **, <0.001 = ***). Inter-group comparisons were made using a one-way analysis of variance (ANOVA) followed by Tukey’s post hoc test for further group comparisons.

## 5. Conclusions

Results obtained from in vitro and in vivo studies confirm the exclusive anti-leukemic potential of nanomedicine, in accordance with in silico studies posing our nanomedicine as a potent inducer of the TRAIL-DR5 complex (pdb:1du3) and inhibitor of an NF-kB p52/RelB/DNA complex. However, further research is required to verify the specificity, targeting, and biocompatibility of this nanomedicine and test it through clinical trials. Although several chemotherapies against acute myeloid leukemia are commercially available, their harmful side effects render them less preferred for treatment. Our study proposes a solution to such limitations, as the efficacy of caffeine against acute myeloid leukemia has been enhanced significantly in the form of a nanomedicine. This further signifies the role of nanoparticles as excellent modulators for increasing the anticancer potential of natural compounds and reducing the harmful side effects of chemotherapy.

## Data Availability

Data is contained within the article and Supplementary Material.

## Supplementary Materials

The following supporting information can be downloaded at: https://www.mdpi.com/article/10.3390/ph16121742/s1, Figure S1: Scanning electron microscopy image of mesoporous silica nanoparticles showing their uniform shape and size with an average diameter of 120 nm. Figure S2: X-ray diffraction analysis of MSNPs using X-ray spectrophotometer (Bucker D8 Advance) and Debye-Scherrer equation with diffraction peak at 2θ = 29.18. Figure S3: FTIR analysis of (a) caffeine (b) MSNPs, and (c) nanomedicine using FTIR spectroscope (Bruker, Tensor 27) with a scan range of 400 to 4000 cm−1 indicating caffeine coating over MSNPs. Figure S4: UV/Vis Spectroscopy absorbance spectra of (a) MSNPs, (b) caffeine, and (c) nanomedicine using UV-VIS spectrophotometer (Perkin Elmer UV/VIS-Lambda 25) within 250–800 nm spectrum confirm caffeine loading over MSNPs. Figure S5: Blood cells morphology of normal (A), leukemic (B), doxorubicin (C), caffeine (D), nanomedicine (E), and nanomedicine in combination with chemotherapy (F) in rats using high-resolution microscope at 100×. The RBC count was decreased, and WBC morphology was distorted with the increased nucleus-to-cytoplasm ratio along with leukocytosis in leukemic cells; however, recovery of normal morphology is observed in treated groups. Figure S6: Comparison of mean + SEM relative organ weights of the liver (A), heart (B), and kidney (C) in different experimental groups. The intergroup comparisons were made using one-way ANOVA with Tukey’s post hoc test. A statistical difference of *p* < 0.05 was taken as significant. Table S1: Dosage regime for Brine Shrimp Assay to assess optimum in-vivo dosage.
