# Supplementary material for: In Silico, In Vitro, and In Vivo Evaluation of Caffeine-Coated Nanoparticles as a Promising Therapeutic Avenue for AML through NF-Kappa B and TRAIL Pathways Modulation
Source: Pharmaceuticals (Basel). 2023 Dec 18;16(12):1742. doi: 10.3390/ph16121742 (PMC10747568; doi:10.3390/ph16121742)
Supplement: Supplementary file 1 [file pharmaceuticals-16-01742-s001.zip › pharmaceuticals-2742055-supplementary.pdf]

Supplementary Figures

Supp. Figure S1:

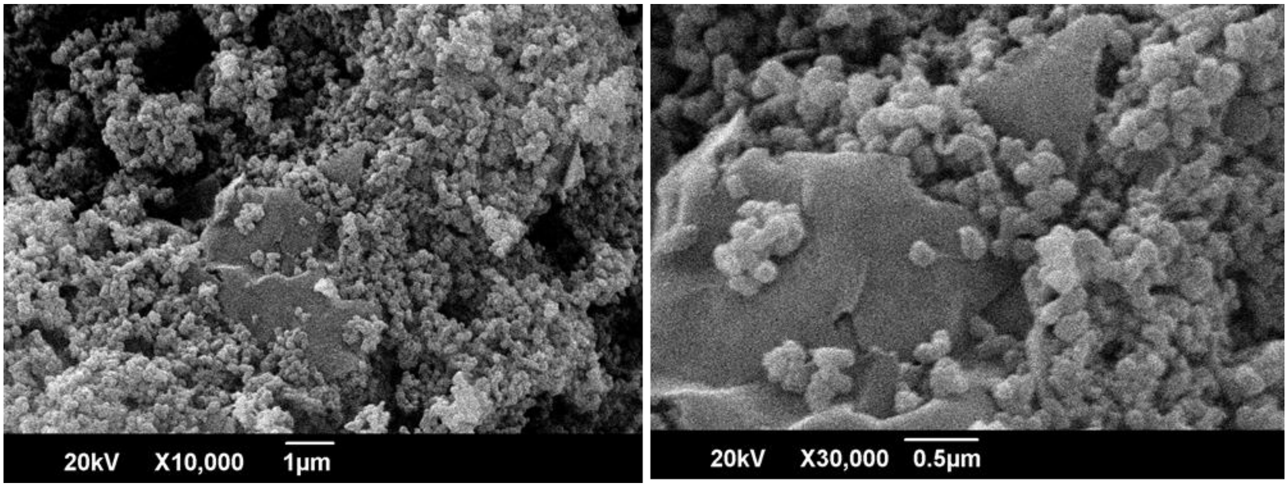

Suppl. Figure S1: Scanning electron microscopy (SEM) images of mesoporous silica nanoparticles showing the honeycomb porous structure with average 120 nm size of particles

Suppl. Figure S2:

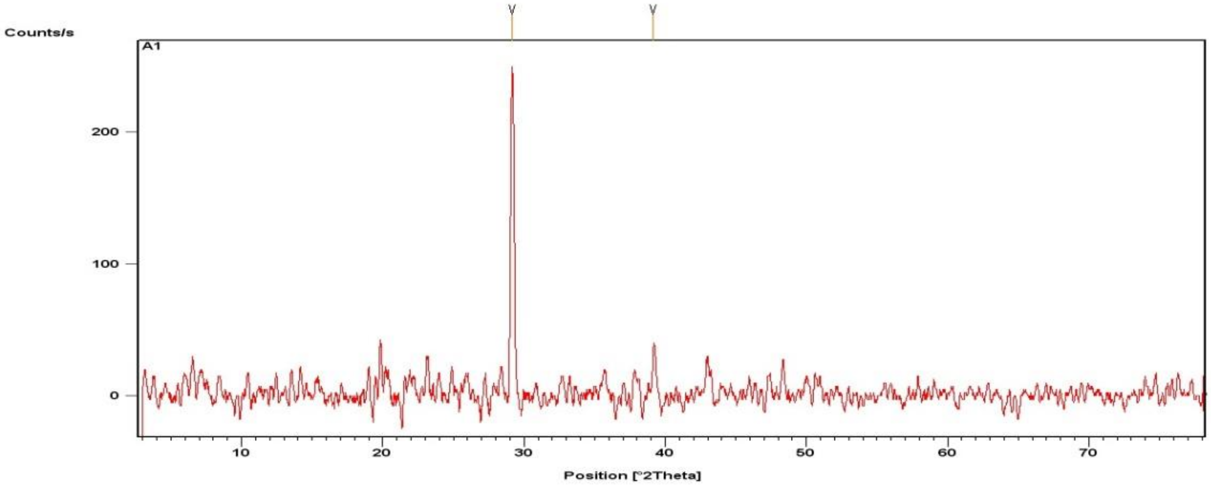

Suppl. Figure S2: X-Ray Diffraction Analysis of MSNPs using X-ray spectrophotometer (Bucker D8 Advance) and Debye Scherrer equation with diffraction peak at 2 TB= 29.18.

Suppl. Figure S3.

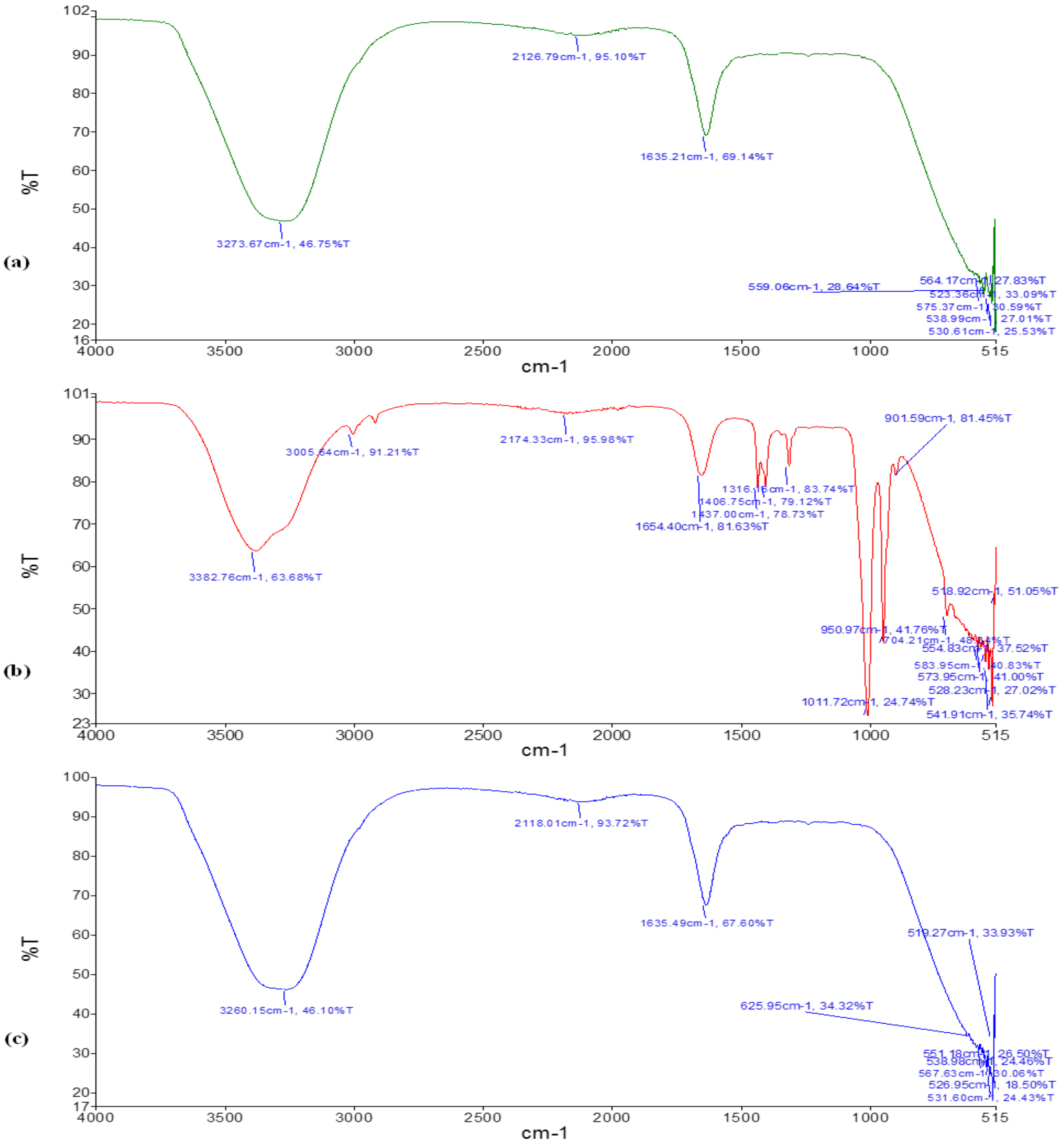

Suppl. Figure S3: FTIR analysis of (a) Caffeine (b) MSNPs and (c) Nanomedicine using FTIR spectroscope (Bruker, Tensor 27) with a scan range of 400 to 4000 cm-1 indicating caffeine coating over MSNPs.

Suppl. Figure S4.

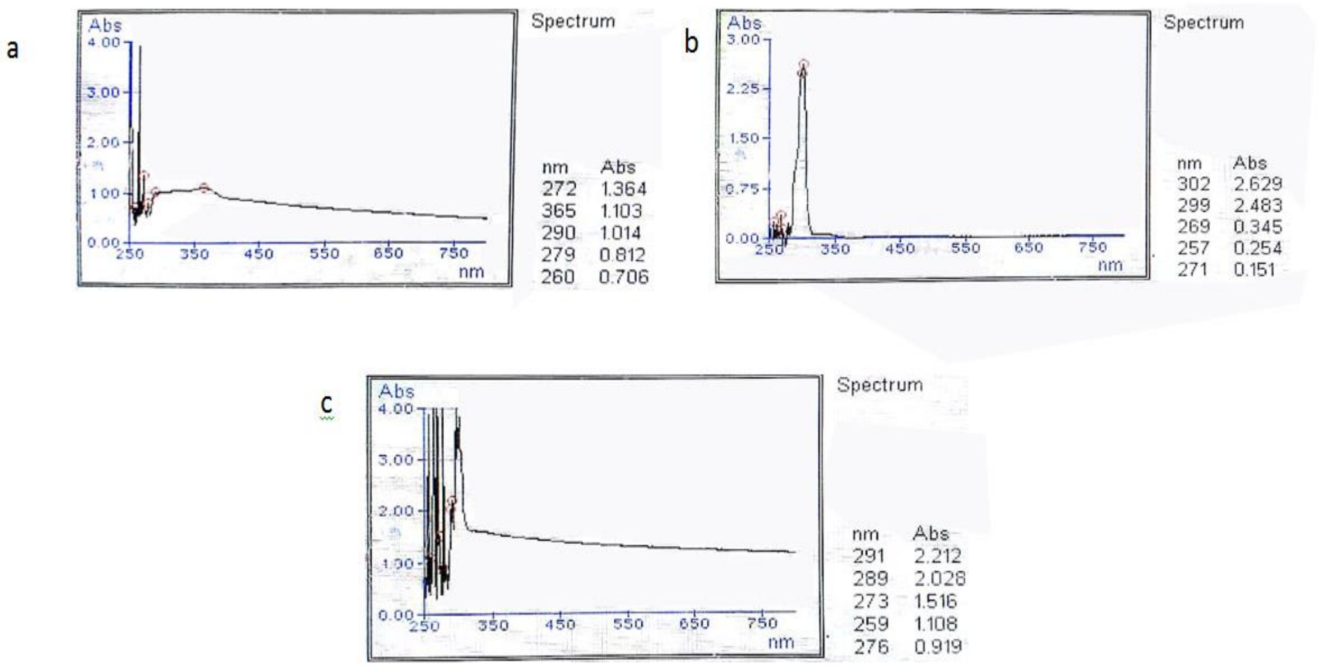

Suppl. Figure S4: UV/Vis Spectroscopy Absorbance spectra of a) MSNPs, b) Caffeine & c) Nanomedicine using UV-VIS spectrophotometer (Perklin Elmer UV/VIS-Lambda 25) within 250-800nm spectrum confirm caffeine loading over MSNPs.

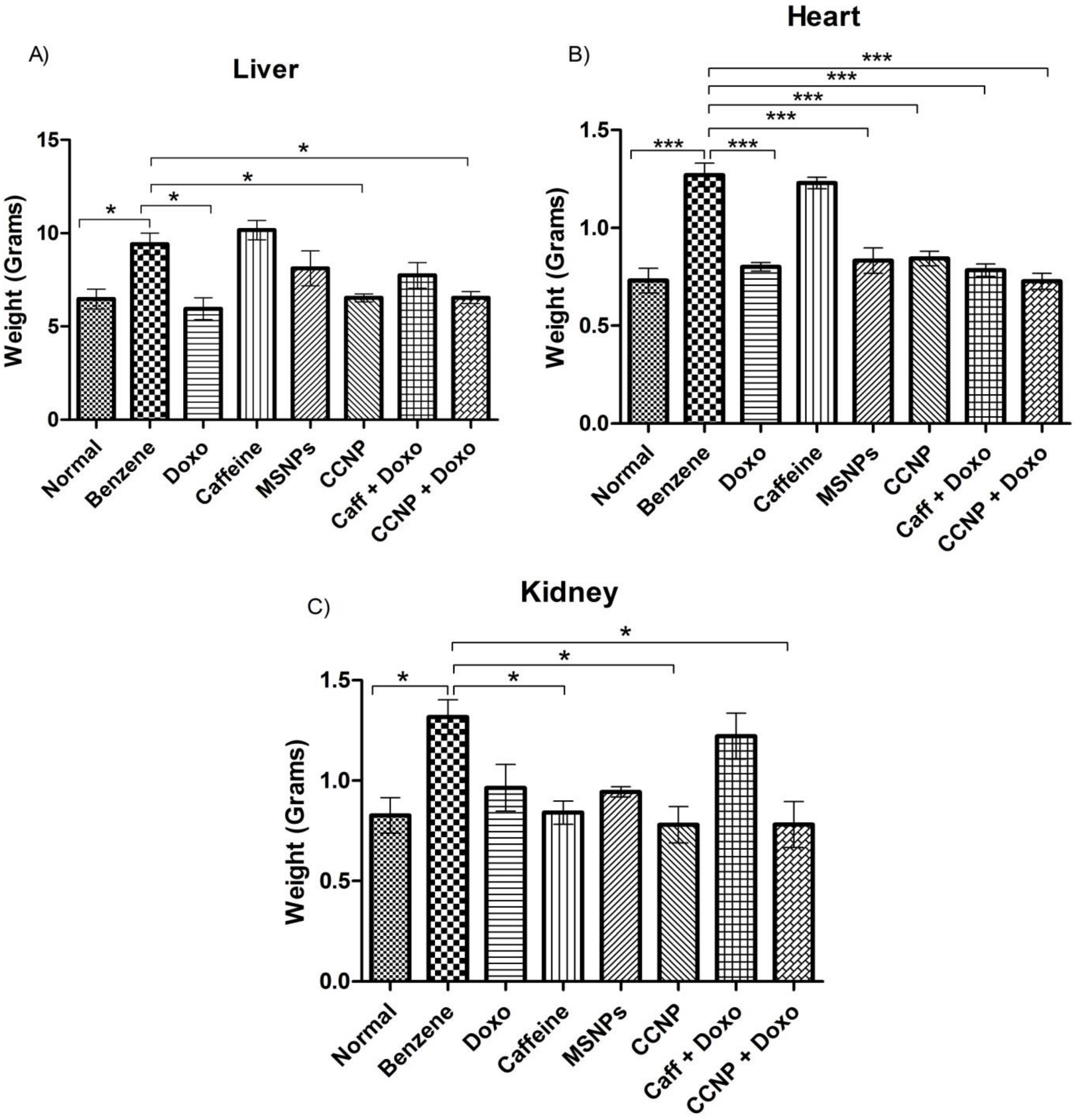

Suppl. Figure S5: Comparison of mean + SEM relative organ weights of liver (A), heart (B) and kidney (C) in different experimental groups. The intergroup comparisons were made using one-way ANOVA with Tukey's post hoc test. Statistical difference of $p<0.05$ was taken as significant.

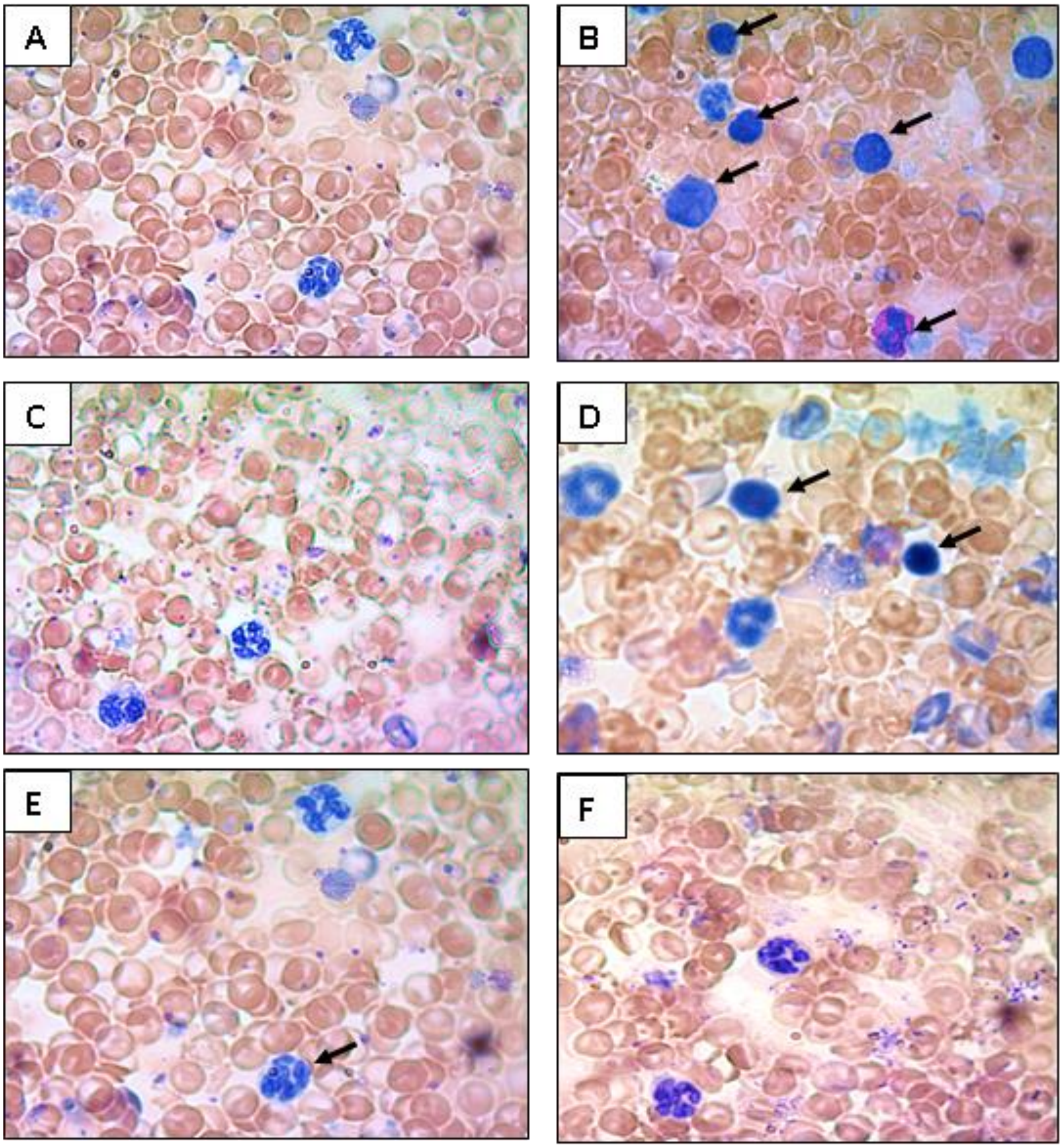

Supp. Figure S6: Blood cells morphology of normal (A) leukemic (B) doxorubicin (C) Caffeine (D) Nanomedicine (E) and Nano-medicine in combination with Chemotherapy (F) in rats using high resolution microscope at 100x. The RBC count was decreased and WBC morphology was distorted with increased nucleus to cytoplasm ratio along with leukocytosis in leukemic cell however, recovery of normal morphology is observed in treated groups.

Supp. Table S1

No.	Drug	Conc. 1	Conc. 2	Conc.3	Conc.4
**1.**	Caffeine	33.4mg/mL	16.7mg/mL	8.35 mg/ml	4.175mg/mL
**2.**	Doxorubicin	2.1mg/mL	1.05mg/mL	0.525mg/ml	0.2625mg/mL
**3.**	Caffeine + Doxorubicin	16.7mg/500ul + 1.05mg/500ul	8.32mg/500ul + 0.525mg/500ul	4.18mg/500ul + 0.26mg/500ul	2.09mg/500ul + 0.13mg/500ul

Suppl. Table S1: Dosage regime for Brine Shrimp Assay to assess optimum in-vivo dosage
